# Contamination. New developments of a corrective bracing concept resulting from a matching with a different approach

**DOI:** 10.1186/1748-7161-7-S1-P6

**Published:** 2012-01-27

**Authors:** F Tessadri, M Tavernaro, A Zonta, S Negrini

**Affiliations:** 1 Orthotecnica, Gardolo di Mezzo, Italy

## Background

The SPoRT (Symmetric, Patient oriented, Rigid, Three-Dimensional) Sforzesco brace has been developed recently and it is continuously in evolution [1].

PurposePresentation of two new developments to improve the SPoRT concept coming from the Cheneau concept of bracing.

## Materials and methods

The excellent performance of the bottom up action of the SPoRT brace is guaranteed by the drivers, a concept first developed with the Sforzesco brace. Since these cannot be present at the brace’s upper and lower margins, it has been hypothesized that the postural action of the Cheneau concept could be used to obtain an increasing of correction. Two innovations (called “Cheneuization” and “open pelvis”) has been introduced and tested in a series of cases, with progressive improvement and final selection for application [2]. During the presentation we will discuss a range of clinical cases and carried out experimentations.

## Results

We have introduced two important innovations following one upon the other: the first one relating to the shoulder girdle and the second one relating to the pelvic girdle. In both cases we have obtained a visible translation sustained from the rigidity of the material as well as from the effects of the proper drivers of the SPoRT Brace, according to the aesthetic and appreciably symmetric SPoRT approach.

In particular, the “Cheneausation” relating to the shoulder girdle has obtained creditable results in the course of two years of experimentation and has been added to the SPoRT concept. The open pelvis, instead, is still in course of study and development.
